# Supplementary figures and images for: Circuit-based intervention corrects excessive dentate gyrus output in the fragile X mouse model
Source: eLife. 2024 Feb 12;12:RP92563. doi: 10.7554/eLife.92563 (PMC10942577; doi:10.7554/eLife.92563)

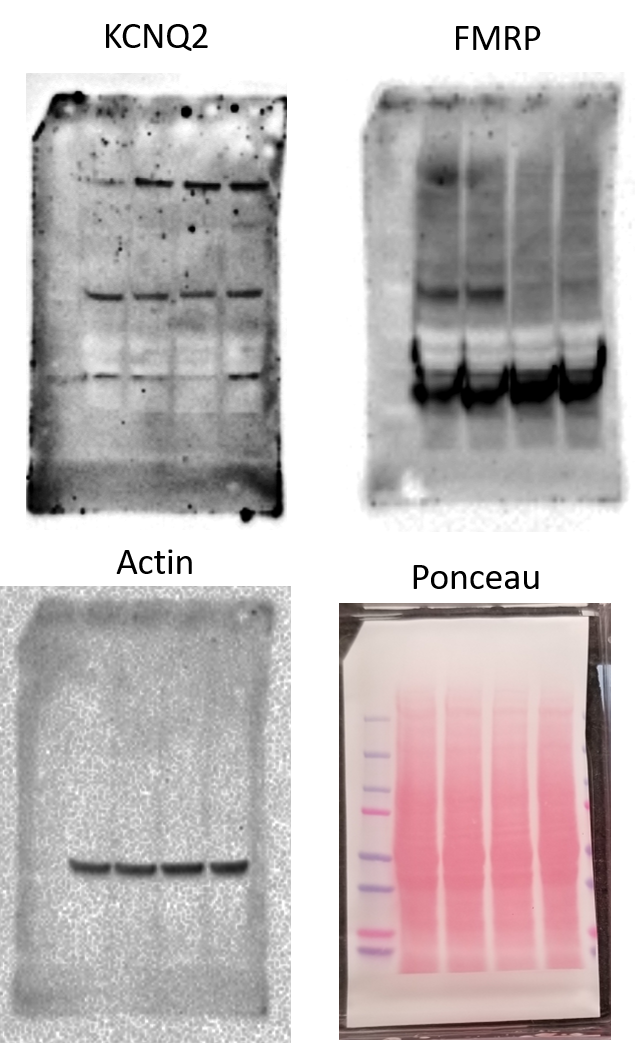

Supplement: Figure 3—figure supplement 1—source data 1. [file elife-92563-fig3-figsupp1-data1.zip › Figure 3 figure supplement 1 source data 1A.tif]

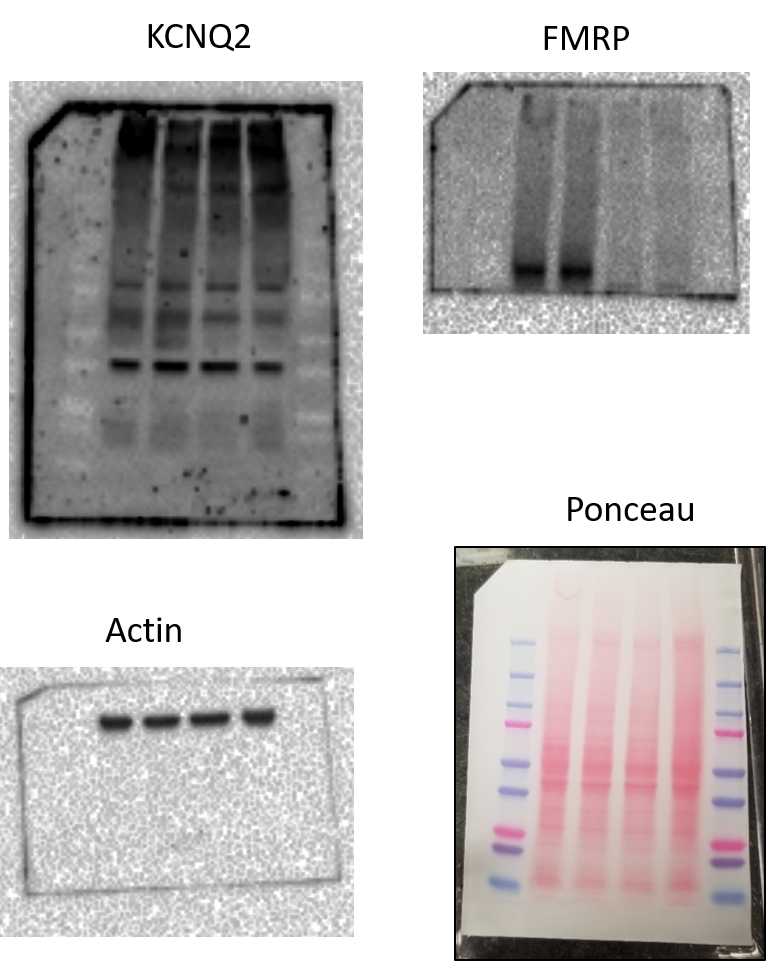

Supplement: Figure 3—figure supplement 1—source data 1. [file elife-92563-fig3-figsupp1-data1.zip › Figure 3 figure supplement 1 source data 1C.tif]

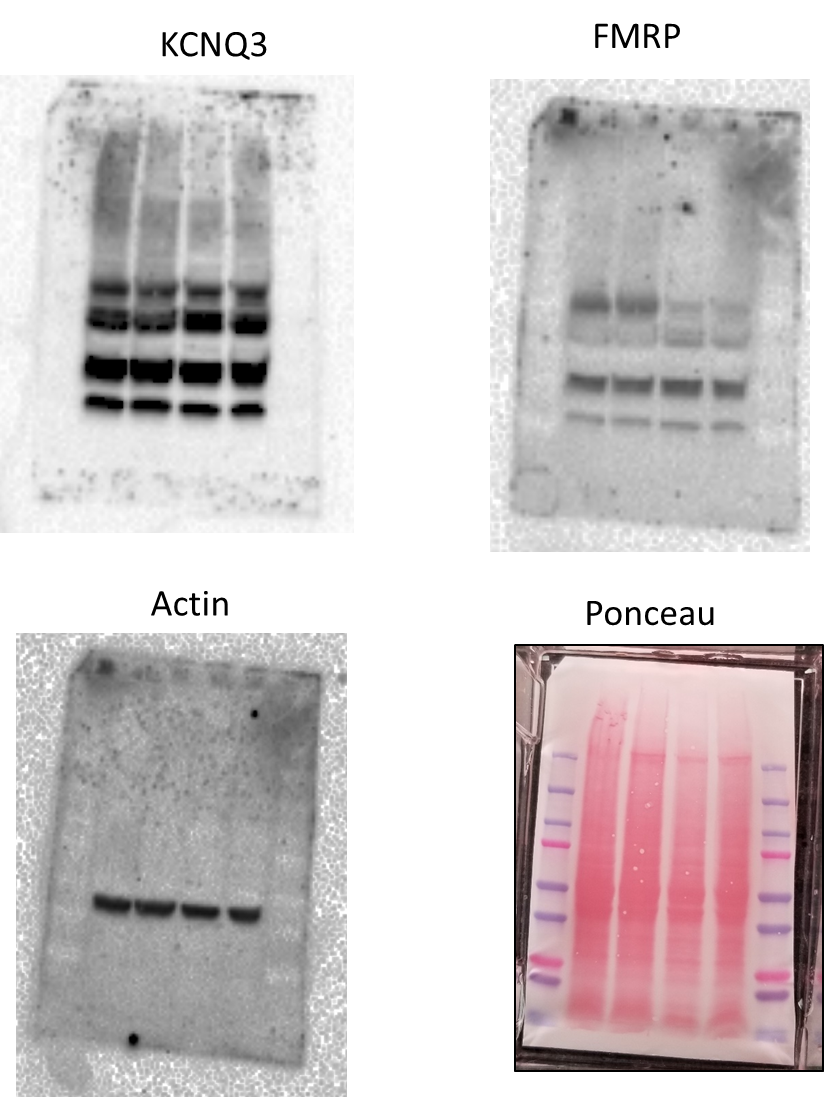

Supplement: Figure 3—figure supplement 1—source data 1. [file elife-92563-fig3-figsupp1-data1.zip › Figure 3 figure supplement 1 source data 1D.tif]

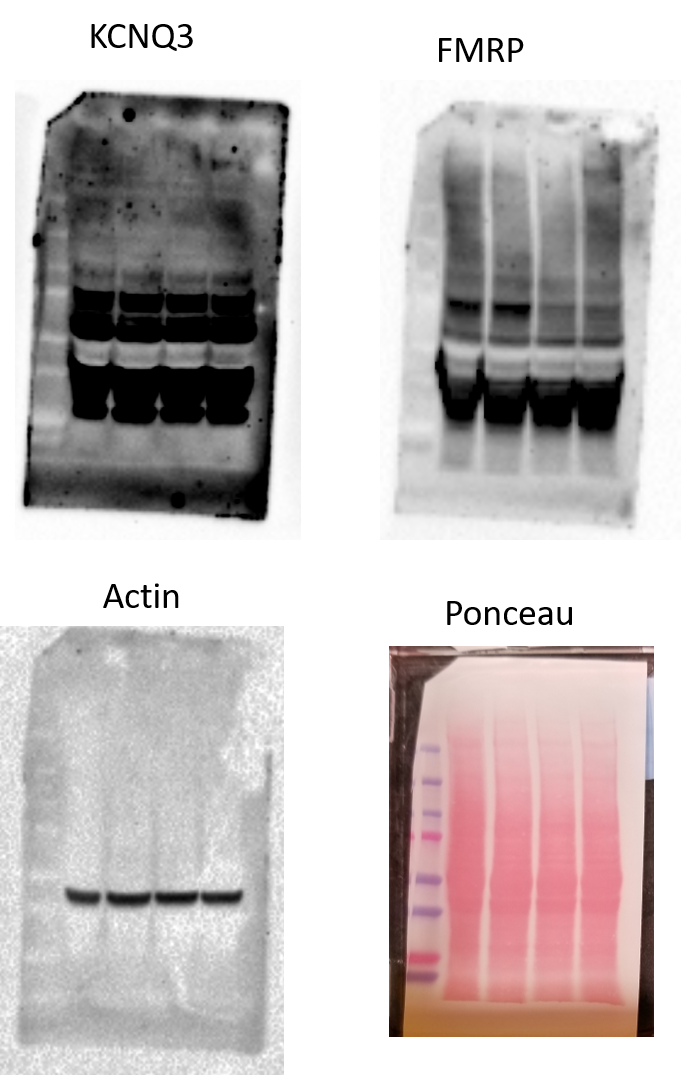

Supplement: Figure 3—figure supplement 1—source data 1. [file elife-92563-fig3-figsupp1-data1.zip › Figure3 figure supplement 1 source data1B.tif]

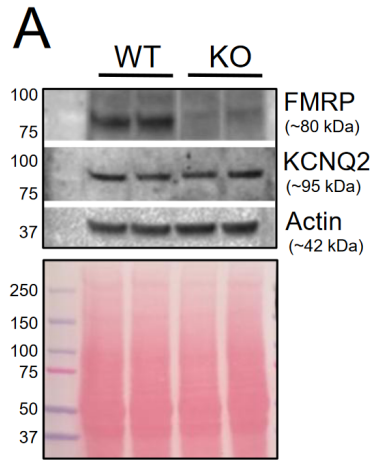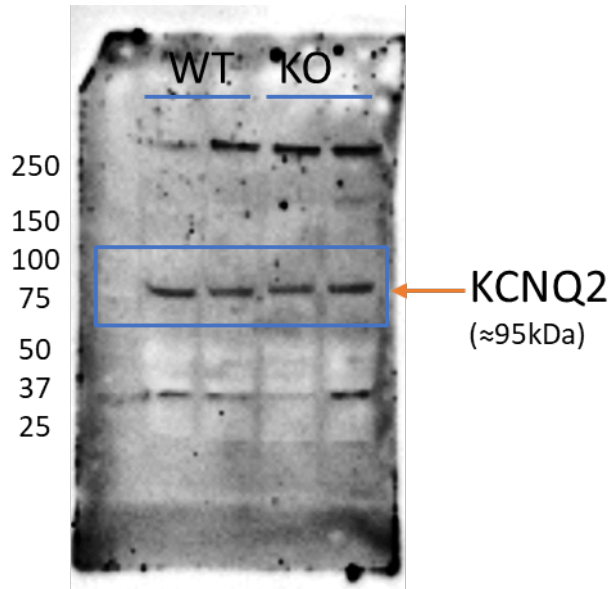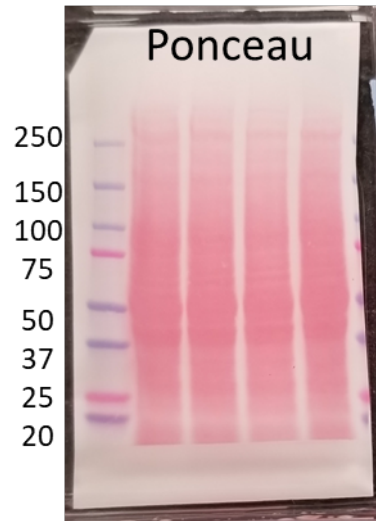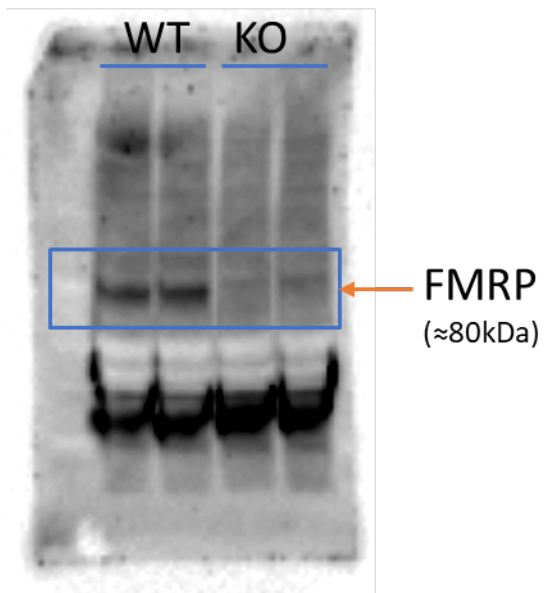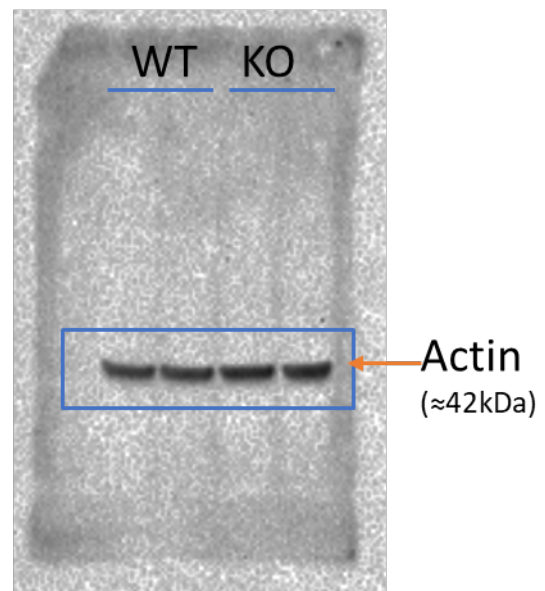

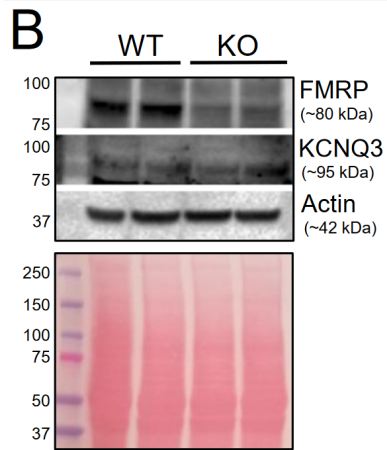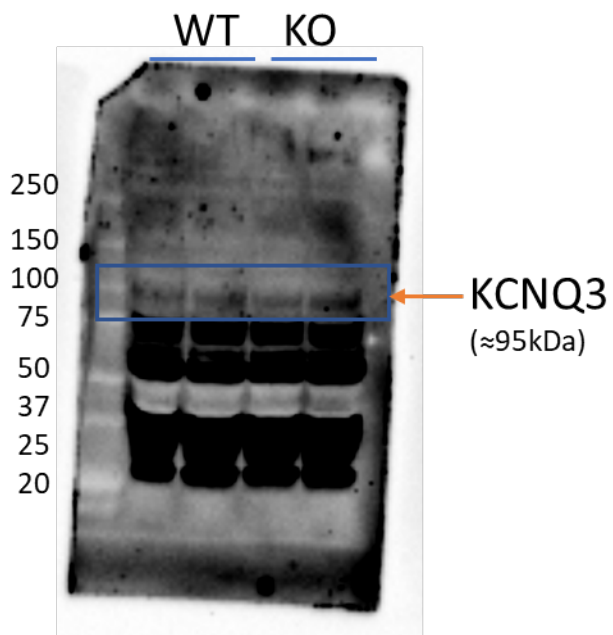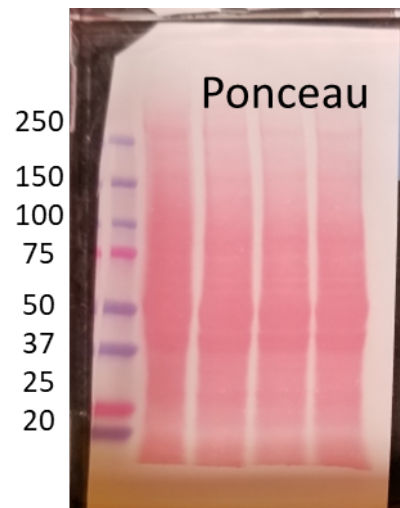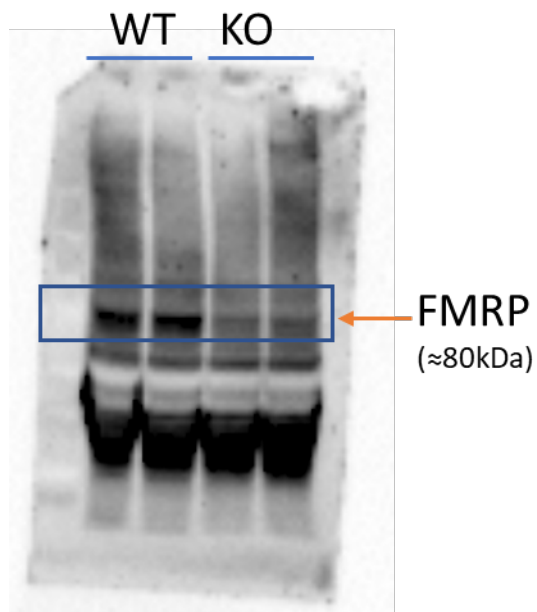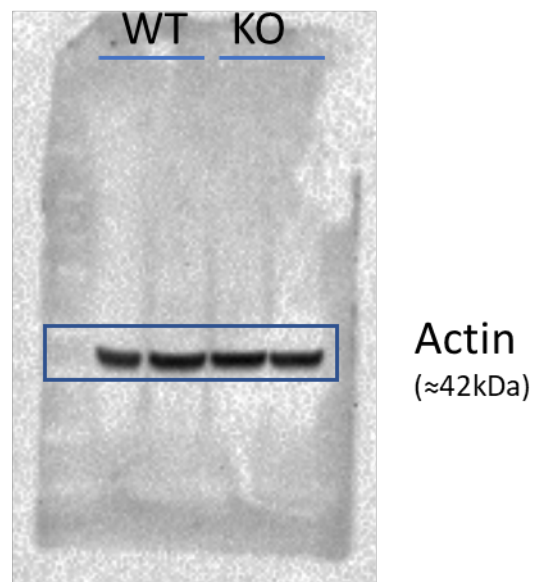

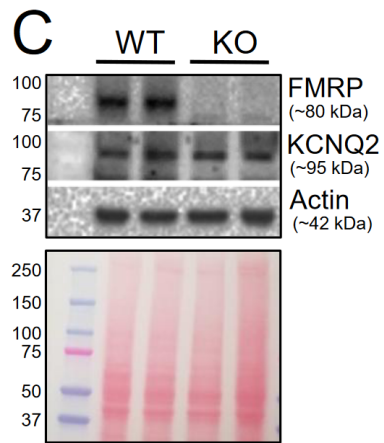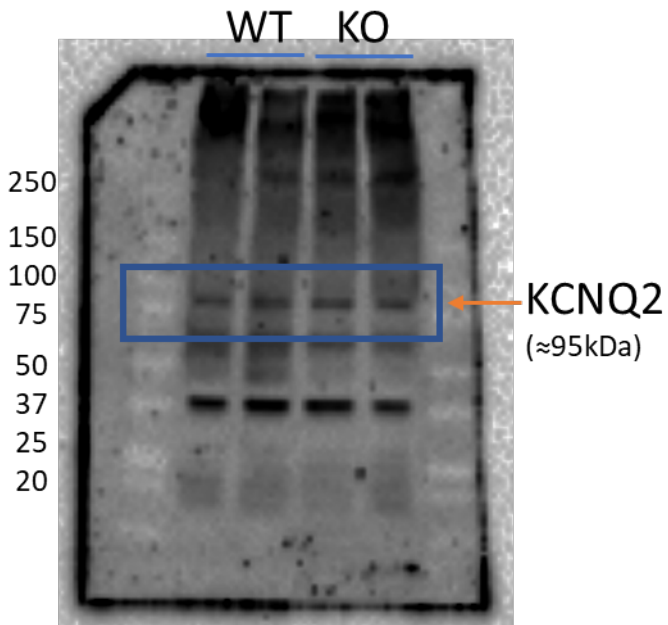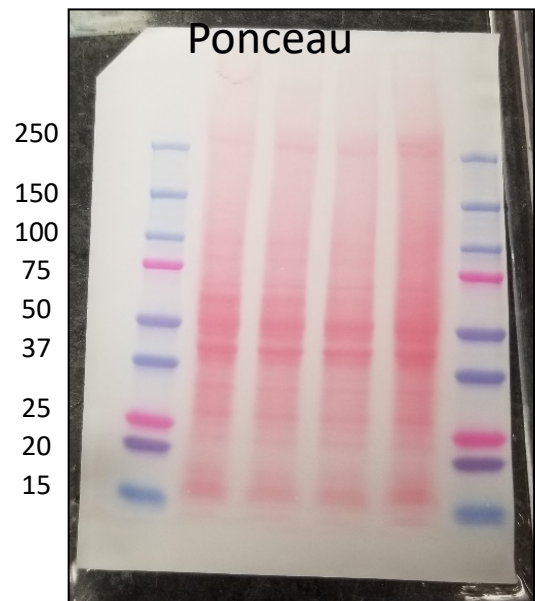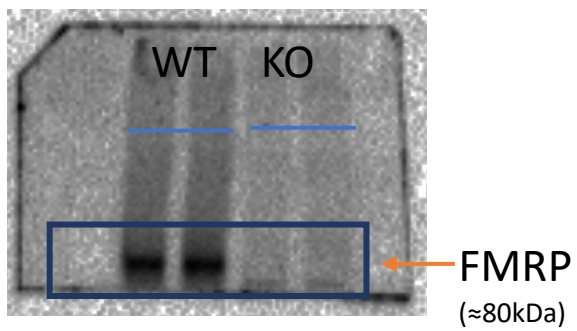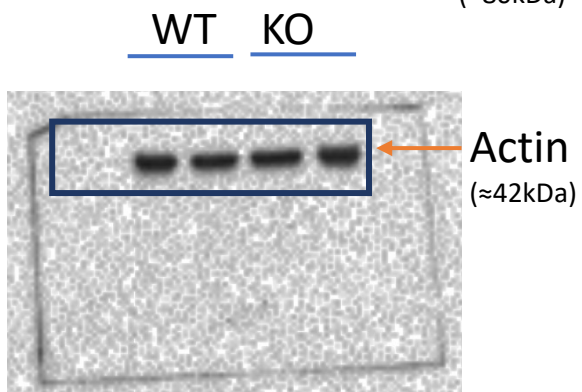

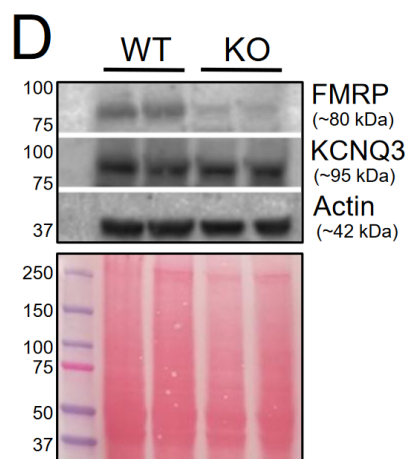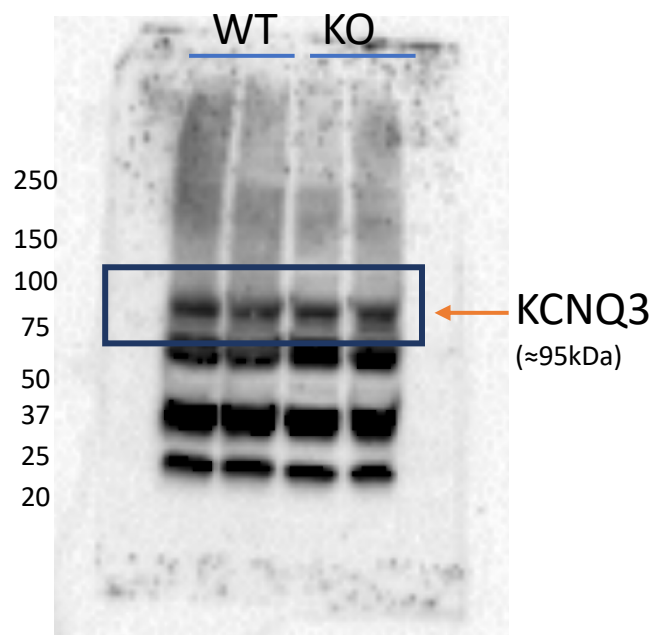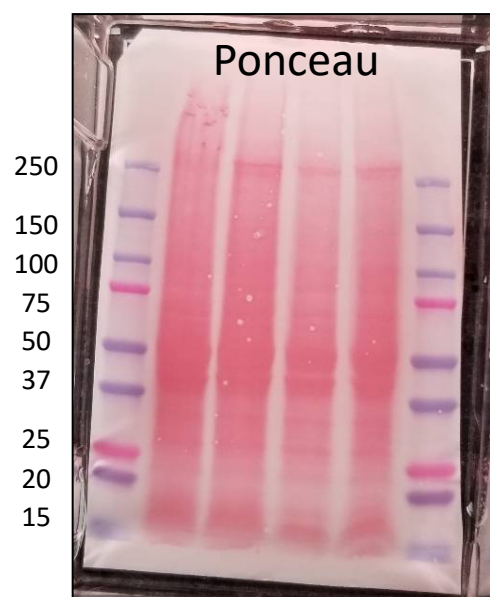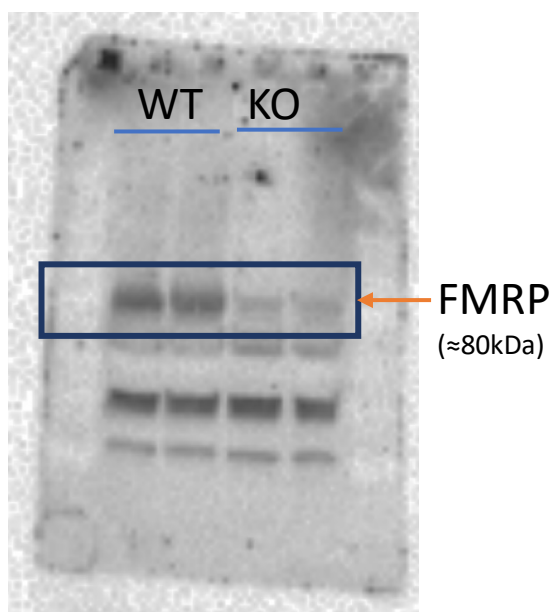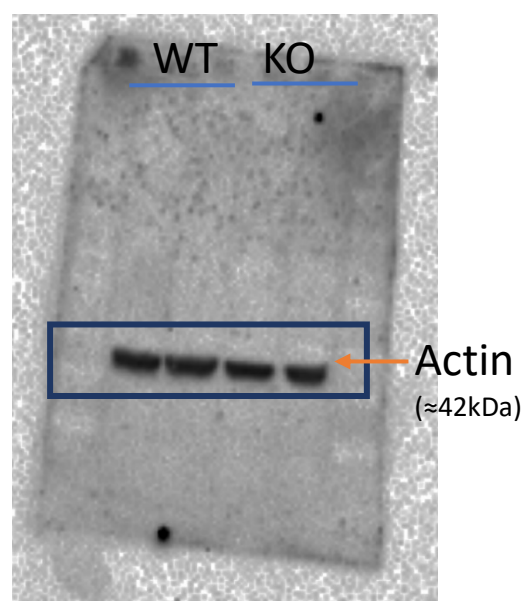

Supplement: Figure 3—figure supplement 1—source data 2. [file elife-92563-fig3-figsupp1-data2.pdf]
